# A Long Short-Term Memory Ensemble Approach for Improving the Outcome Prediction in Intensive Care Unit

**DOI:** 10.1155/2019/8152713

**Published:** 2019-11-03

**Authors:** Jing Xia, Su Pan, Min Zhu, Guolong Cai, Molei Yan, Qun Su, Jing Yan, Gangmin Ning

**Affiliations:** ^1^Department of Biomedical Engineering, Zhejiang University, 38 Zheda Road, Hangzhou 310027, China; ^2^Department of ICU, Zhejiang Hospital, 12 Lingyin Road, Hangzhou 310013, China; ^3^Department of ICU, The First Affiliated Hospital, Zhejiang University, 79 Qingchun Road, Hangzhou 310003, China

## Abstract

In intensive care unit (ICU), it is essential to predict the mortality of patients and mathematical models aid in improving the prognosis accuracy. Recently, recurrent neural network (RNN), especially long short-term memory (LSTM) network, showed advantages in sequential modeling and was promising for clinical prediction. However, ICU data are highly complex due to the diverse patterns of diseases; therefore, instead of single LSTM model, an ensemble algorithm of LSTM (eLSTM) is proposed, utilizing the superiority of the ensemble framework to handle the diversity of clinical data. The eLSTM algorithm was evaluated by the acknowledged database of ICU admissions Medical Information Mart for Intensive Care III (MIMIC-III). The investigation in total of 18415 cases shows that compared with clinical scoring systems SAPS II, SOFA, and APACHE II, random forests classification algorithm, and the single LSTM classifier, the eLSTM model achieved the superior performance with the largest value of area under the receiver operating characteristic curve (AUROC) of 0.8451 and the largest area under the precision-recall curve (AUPRC) of 0.4862. Furthermore, it offered an early prognosis of ICU patients. The results demonstrate that the eLSTM is capable of dynamically predicting the mortality of patients in complex clinical situations.

## 1. Introduction

Mortality prediction is essential for the clinical administration and treatment, especially in the intensive care unit (ICU) [1, 2]. Various scoring systems have been developed and widely used for assessing the clinical outcome, and the most common ones are simplified acute physiology score (SAPS) II [3], sequential organ failure assessment (SOFA) [4], and acute physiology and chronic health evaluation (APACHE) II [5]. Scoring systems assess the patients' mortality by logistic regression model assuming a linear and addictive relationship between the severity of the disease and the collected relevant physiological parameters, which are practicable but unrealistic [6]. In the recent years, machine learning was introduced in the medical application and showed its remarkable efficiency in clinical diagnosis and decision support. For admitted ICU patients, lots of physiological measurements are collected, containing symptoms, laboratory tests, and vital signs (such as heart rate, blood pressure, and respiratory rate) [7, 8]. The clinical measurements are continuously monitored in ICU with the values fluctuating as time progresses and the temporal trends are predictive of mortality [9]. Hence, sequence of clinical records offers rich information of patients' physical condition [10, 11] and enables the utilization of machine learning in developing prognosis model from these multivariate time series data. As a decision task, mortality prediction can be solved by classification algorithms such as logistic regression, support vector machine, and random forests (RF) [12]. However, most of the methods currently used are not sensitive to the temporal link among the sequent data and thus are not able to receive full benefits of the ICU data, which limits their performances in the mortality prediction [10, 13].

Presently, recurrent neural network (RNN) was well employed in solving time series prediction problems and achieved prominent results in many fields [14–19]. Several variants of RNN have been developed, and among them, long short-term memory (LSTM) network is one of the most popular variants [20]. LSTM learns long-term dependencies by incorporating a memory cell that is able to preserve state over time. Three gates are equipped in LSTM for deciding which information to summarize or forget before moving on to the next subsequence [21–23]. LSTM is well suited to capture sequential information from temporal data and has shown advantages in machine translation [24, 25], speech recognition [19], and image captioning [26], etc. In the medical domain, many efforts have been made to apply LSTM for clinical prediction based on electronic health records [6, 17, 27–30]. Lipton et al. employed LSTM on a collection of 10, 401 episodes to establish a model for phenotype classification [28]. Given 13 frequently sampled clinical measurements (diastolic and systolic blood pressure, peripheral capillary refill rate, end-tidal CO_2_, fraction of inspired O_2_, Glascow coma scale, blood glucose, heart rate, pH, respiratory rate, blood oxygen saturation, body temperature, and urine output), the LSTM model was able to predict whether the patient suffered from 128 most common conditions, such as acute respiratory distress, congestive heart failure, and renal failure. Jo et al. used LSTM and latent topic model to extract information from textual clinical notes for assessing the severity of diseases [29]. Pham et al. conducted experiments on a diabetes cohort of 7191 patients with 53208 admissions collected in 2002–2013 from a large regional Australian hospital, and the results showed improved performances of utilizing LSTM in disease progression modeling and readmission prediction [31].

For ICU mortality prediction, the current prognosis models mostly employed single LSTM classifier [6, 29, 30]. However, in most cases, a single model is not efficient enough to handle the complex situation in ICU. Patients in ICU are heterogeneous suffering from different diseases with multiple concurrent problems, and the clinical data in ICU are highly complex [9, 32, 33]. For patients with various diseases, the underlying pathophysiologic evolutions of the patients (e.g., kidney failure) are usually manifested through different sets of physiologic variables (e.g., abnormalities in glomerular filtration rate and creatinine) [9]. Even for the patients having the same disease, they might have different comorbidities experiencing heterogeneous health conditions [33]. Thereby, hybrid learners are required for the prediction model in ICU.

An ensemble learner principally has a stronger generalization ability than a single learner [34–37]. Ensemble learning is a procedure that integrates a set of models for a given problem to obtain one composite prediction [38–43]. Diverse classifiers are constructed to learn multiple hypotheses, and the multiple resulting predictions are aggregated to solve the same problem. In contrast to the stand-alone model which builds one hypothesis space, a combination of several models can expand the space and may provide a more exact approximation to the true hypothesis [34]. It has been shown that ensemble systems outperformed single classifier systems in solving complex problems [34, 38, 39].

Therefore, we proposed an ensemble algorithm of multiple long short-term memory networks (eLSTMs) to deal with the complex situation in ICU. In eLSTM, the diversity of LSTM models owes to the multifariousness of subsets for building the models. Two strategies are employed to produce different subsets from the entire training data, namely, bootstrapped samples and random feature subspace. Bootstrapped samples strategy generates various subsets of subjects, while random feature subspace provides different combined sets of clinical indicators. That is, the subsets are distinguished from each other at both instance and feature level. A variety of LSTM classifiers are trained accordingly, and the final score is computed as the average of predicted values from all base learners. Generally, the eLSTM algorithm selects a number of training subsets using bootstrapped instances with randomly chosen feature set, constructs multiple LSTM learners on the multiple subsets, and averages all individuals' predicted scores as final output. The main contributions of this work are as follows: (1) proposing an LSTM ensemble framework to develop hybrid sequential classification model which is able to handle complex clinical situations such as ICU and (2) applying bootstrapped samples and random feature subspace to individual LSTM classifiers for creating diversity in the ensemble. The present model will promote the application of machine learning in complex clinical situations.

The rest of this paper is organized as follows.  Section 2 describes the ICU dataset, the implementation of the proposed eLSTM algorithm, and the experimental design. The empirical results yielded by various systems for mortality prediction are presented in Section 3. The advantages of eLSTM are discussed in Section 4. Finally, Section 5 concludes this paper and indicates the future work.

## 2. Methods

### 2.1. Dataset

The ICU data for this work were extracted from the Medical Information Mart for Intensive Care III (MIMIC-III) database [44]. MIMIC-III is a large and publicly available database of ICU admissions at the Beth Israel Deaconess Medical Center, USA, from 2001 to 2012. It comprises rich clinical data of patients, including the laboratory tests and vital signs. A total of 18415 patients were extracted from MIMIC-III database with age >15 years and length of stay ≥10 days. The prediction task of clinical outcome is 28-day postadmission mortality. The study population consists of 2162 subjects in positive group that died within 28 days after ICU admission and the other 16253 subjects in negative group that survived 28 days after ICU admission. From the tables LABEVENTS.csv and CHARTEVENTS.csv, 50 variables of continuous 10 days (denoted as *D*1, *D*2,…, *D*10) are recorded for mortality prediction. The variables are sampled every 24 hours. These variables are commonly used clinical measurements, and the details are listed in Table 1.

### 2.2. LSTM Ensemble Algorithm

Ensemble methods generate multiple learners and aggregate them to provide a composite prediction. Among them, the Bagging and Boosting method are most popular. The diversity of individual learner is an important issue for ensemble model, which can be achieved by selecting and combining the training examples or the input features, injecting randomness into the learning algorithm [34, 36].

The proposed eLSTM algorithm is an ensemble method utilizing LSTM as base learner. Two random strategies are employed to produce different training subsets, hence constructing a number of base LSTM classifiers. All predictions are integrated to give a comprehensive estimate of the outcome.

Given a training set with *N* training instances, each instance can be represented as (*V*, *Y*). *V* is a matrix containing values of *D* variables and *T* sequences. It can be written as [*X*_1_, *X*_2_, *X*_3_,…, *X*_*t*_,…, *X*_*T*_], as expressed in equation (1). *X*_*t*_ is a vector given in equation (2). *x*_*t*_^*d*^ represents the value of the *d*-th variable at *t*-th time step. And *Y* is the target label for the instance taking 0 (negative) for survival and 1 (positive) for death. The ratio of negative and positive group size is denoted as *γ*:

(1) $$\begin{array}{c} V=\left[X_{1},X_{2},X_{3},\ldots ,X_{t},\ldots ,X_{T}\right], \end{array}$$

(2) $$\begin{array}{c} X_{t}=\left[x_{t}^{1},x_{t}^{2},x_{t}^{3},\ldots ,x_{t}^{d},\ldots ,x_{t}^{D}\right]. \end{array}$$

**Figure 1:**
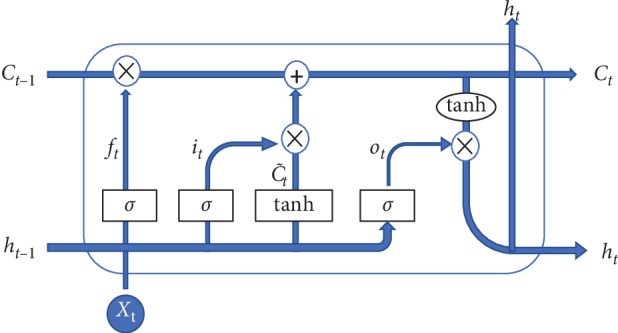
Illustration of the LSTM block's structure.

LSTM has the advantage of capturing temporal information and is popular to be adopted in time series modeling. Detailed structure of the LSTM block is illustrated in Figure 1.

The input of LSTM block is *X*_*t*_. Then, the output of hidden layer, namely, the current hidden state *h*_*t*_, is computed as follows:

(3) $$\begin{array}{c} f_{t}=\sigma \left(w_{f}\left[h_{t-1},X_{t}\right]+b_{f}\right),\\ i_{t}=\sigma \left(w_{i}\left[h_{t-1},X_{t}\right]+b_{i}\right),\\ o_{t}=\sigma \left(w_{o}\left[h_{t-1},X_{t}\right]+b_{o}\right),\\ C_{t}=f_{t}*C_{t-1}+i_{t}*\tanh\left(w_{c}\left[h_{t-1},X_{t}\right]+b_{c}\right),\\ h_{t}=o_{t}*\tanh\left(C_{t}\right), \end{array}$$

where *f*_*t*_, *i*_*t*_, and *o*_*t*_ are the forget, input, and output gates, respectively. *h*_*t*−1_ is the previous hidden state. *C*_*t*−1_ and *C*_*t*_ are previous and current cell memories. The weight matrices *w*_*f*_, *w*_*i*_, *w*_*o*_, and *w*_*c*_ and the bias vectors *b*_*f*_, *b*_*i*_, *b*_*o*_, and *b*_*c*_ are model parameters. The symbol *σ* is the sigmoid function and tanh hyperbolic tangent function. The symbol · denotes matrix multiplication and ^*∗*^ elementwise product.

A sigmoid layer is applied on the output of the LSTM block at final step for binary classification. The predicted score $\tilde{y}$ is computed as equation (4). The loss function is the weighted cross entropy of real label and predicted score $\tilde{y}$ with positive instances weighted *γ* and negative ones weighted 1. The parameters within the net are updated over several iterations to reach the minimum loss value:

(4) $$\begin{array}{c} \tilde{y}=\sigma \left(w_{ho}\cdot h_{T}+b_{ho}\right). \end{array}$$

**Figure 2:**
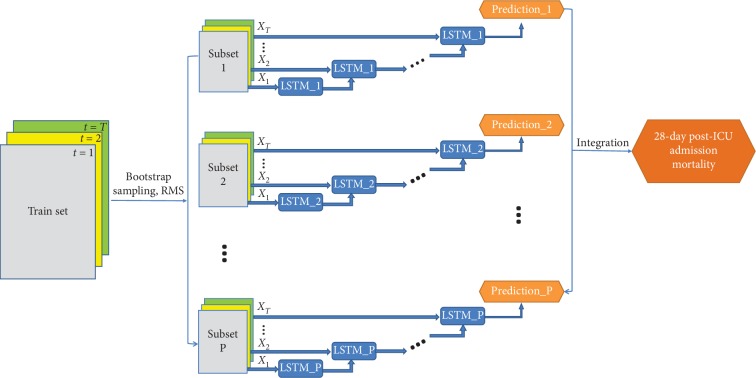
The architecture of the eLSTM algorithm.

The eLSTM model is composed of multiple LSTM classifiers, and its architecture is illustrated in Figure 2.

The procedure of eLSTM consists of two stages: base learner generation and integration.

In the stage of base learner generation, the bootstrap sampling strategy [37] and random subspace method (RSM) [35] are both employed to generate different training subsets for constructing diverse base learners. As a training set sampling method, bootstrap sampling randomly draws instances with replacement from the whole training set and RSM is to randomly choose a subset of variables. The subsets resulted from different bootstrapped instances with randomly selected variables are denoted as {Subset_1_, Subset_2_,…, Subset_*p*_,…, Subset_*P*_}.

In ensemble model rather than error control strategy, bias control is generally adopted to train multiple base classifiers benefiting the diversity of the model. Thus, appropriate number of training epochs for the classifiers is selected by experiments under a satisfied level of bias. The variance of the model due to the diversity of individual classifiers is controlled by the following ensemble operation [45, 46]. For eLSTM, the number of training epochs was set as 100, which was validated by pre-experiments.

Then, multiple LSTM classifiers learn from the subsets. Let {*F*^1^, *F*^2^,…, *F*^*P*^} denote the set of *P* trained base classifiers. For the input *V*, the *p*-th LSTM classifier gives an individual predicted score $\tilde{y}\left(p\right)$, as expressed in equation (5).

Finally, in the integration stage, the scores of all LSTM classifiers are averaged as the overall output and calculated as follows:

(5) $$\begin{array}{c} \tilde{y}\left(p\right)=F^{p}\left(V\right), \end{array}$$

(6) $$\begin{array}{c} \tilde{Y}=\frac{1}{P}\overset{P}{\underset{p=1}{\sum }}\tilde{y}\left(p\right). \end{array}$$

**Figure 3:**
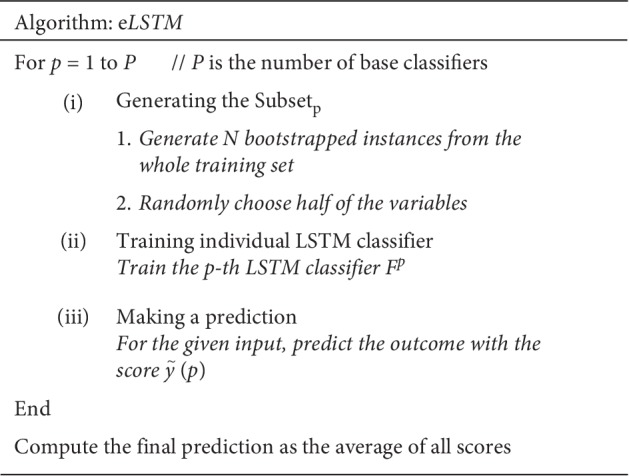
Procedure of eLSTM algorithm.

The procedure of the eLSTM algorithm is provided in Figure 3.

Once the eLSTM model is accomplished, it is applied in this way: for an instance, each LSTM classifier uses partial values of the corresponding variable subset and makes a prediction; different LSTM classifiers utilize different sets of variables, producing multiple prediction scores; the final prediction is obtained by averaging all scores.

### 2.3. Dynamic Prediction

**Figure 4:**
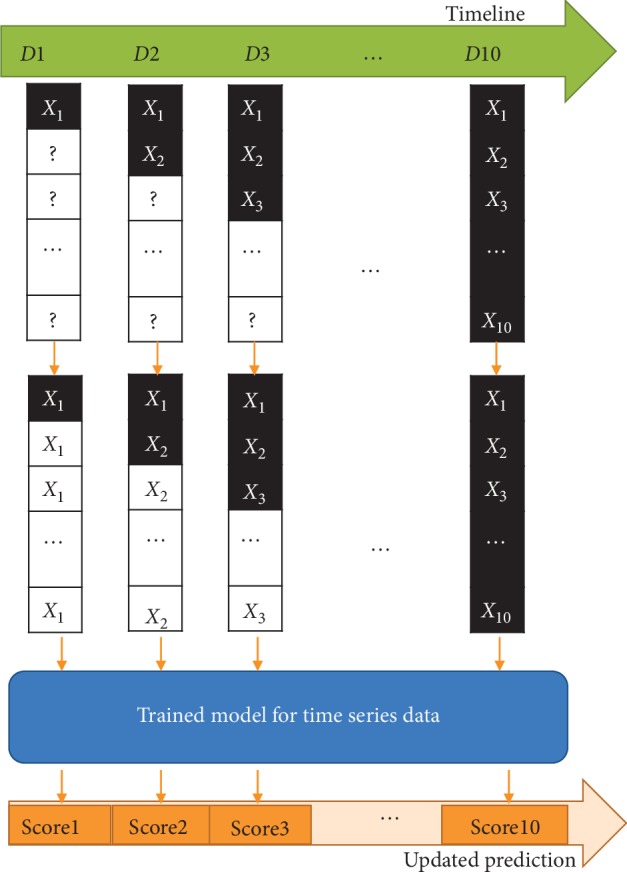
Flow diagram of dynamic prediction with data updating.

For LSTM and eLSTM models, the full sequence of data is needed to predict the outcome. However, in practice, the patients' physiological parameters are collected day by day. To develop a dynamic procedure providing daily prediction, in this work, the values for coming days are padded by the latest available data to acquire complete sequences. Then, the LSTM algorithm and the eLSTM algorithm are employed on the complete dataset for predicting the outcome. Thus, the mortality assessment is updated daily with the replenished data approaching closer to the reliability. The process is illustrated in Figure 4.

### 2.4. Experiment Design

The proposed eLSTM algorithm is compared with three scoring systems (SAPS II, SOFA, and APACHE II), RF algorithm, and LSTM classifier. In the LSTM classifier, a sigmoid layer is applied on top of the LSTM block for binary classification. The LSTM block has one hidden layer with 64 hidden units, and a dropout of rate 0.5 is applied to the input layer. The weight parameters are initialized randomly using Glorot uniform initialization [47]. The LSTM model is trained with the Adam optimizer of learning rate of 0.01 for a maximum of 100 epochs. 10% of the training data are used as a validation set to find the best epoch. In eLSTM algorithm, there are two important hyperparameters: the number of base LSTM classifiers and the size of variable subset. Considering the running time, the number of base LSTM classifiers in the current work is set as 200. And, half of the variables are randomly chosen to construct individual classifier as recommended in the literature [35]. Eventually, 200 individual LSTM-based classifiers are trained on resampled instances with 25 randomly selected variables. In addition, dynamic prediction by RF algorithm is realized by training 10 models on data of the first 1, 2,…, 10 days, respectively.

The experiment is repeated 50 times. For each experiment, 90% of the dataset is chosen as training data and the left 10% as test data. Before the training procedure, data are preprocessed by imputation and normalization. The missing values are filled by linear interpolation imputation method, assuming a linear development in time of the variable with missing data [48]. Then, all the variables are normalized by subtracting the means and dividing the standard deviations computed across the training data.

To compare the performances of these models, several metrics are computed on predicted scores and true labels. The receiving operating characteristics (ROC) curve and the precision-recall curve are plotted to evaluate the performance of the classifiers. The ROC curve uses 1 − specificity as the *x*-axis and sensitivity as the *y*-axis for all potential thresholds, while the precision-recall plot applies recall and precision as the *x*-axis and *y*-axis. The area under ROC (AUROC) and the area under precision-recall curve (AUPRC) are calculated for comparison. Moreover, the bias between the predicted class labels and the true labels is comprehensively measured by sensitivity/recall, specificity, accuracy, precision, and F1 score. Sensitivity/recall calculates how many true-positive cases are correctly classified as positive, while precision counts the proportion of true-positive cases in the cases classified as positive. F1 score is the harmonic mean of recall and precision.

## 3. Results

### 3.1. Mortality Prediction Performance

**Figure 5:**
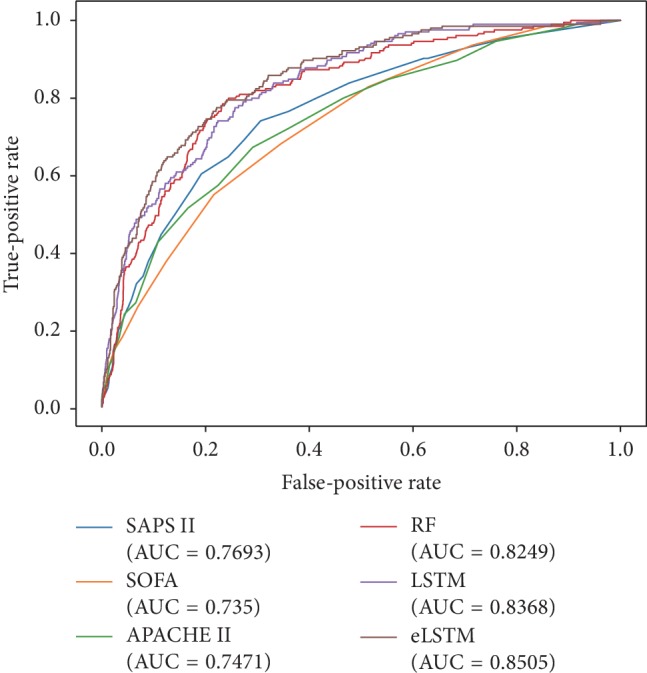
The ROC curves of all systems.

**Figure 6:**
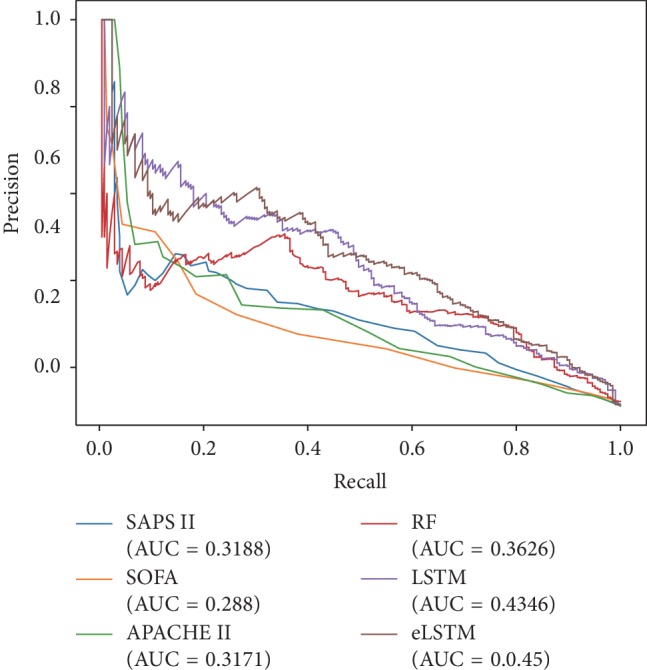
The precision-recall curves of all systems.

The ROC curves and precision-recall curves of all models are shown in Figures 5 and 6. The eLSTM model harvests the largest AUROC of 0.8505 and the largest AUPRC of 0.45.

Detailed statistical results of repeated experiments are given in Table 2. ANOVA test shows significant differences in AUROC, AUPRC, sensitivity/recall, specificity, accuracy, precision, and F1 among the utilized methods (*p* < 0.001). It can be seen the models of RF, LSTM, and eLSTM have much larger AUROC values (RF: 0.8282 ± 0.0151, LSTM: 0.8382 ± 0.0158, and eLSTM: 0.8451 ± 0.0136) than scoring systems SAPS II, SOFA, and APACHE II (SAPS II: 0.7788 ± 0.0166, SOFA: 0.7354 ± 0.0184, and APACHE II: 0.7467 ± 0.0173). The proposed eLSTM model has the largest mean AUROC value of 0.8451, LSTM approach the second largest mean AUROC value of 0.8382, and the RF method the third largest of 0.8282. The eLSTM model outperforms other models in terms of AUPRC with the largest value of 0.4862 ± 0.0345. Also, the eLSTM algorithm has the largest sensitivity/recall of 0.7758 and the RF model and LSTM model have the medium value of 0.7197 and 0.7384, while the three scoring systems get the least value of 0.5418–0.6922. Post hoc analysis by Dunnett test shows the differences in AUROC, AUPRC, and sensitivity between eLSTM and other methods are significant (*p* < 0.05). Totally, the eLSTM model obtains the significant largest value of AUROC, AUPRC, and sensitivity. It is noticed that all methods have low precision and F1 score. It is mainly due to the imbalanced distribution of class label, that is, the number of negative instances is much larger than that of positive ones.

### 3.2. Dynamic Prediction

**Figure 7:**
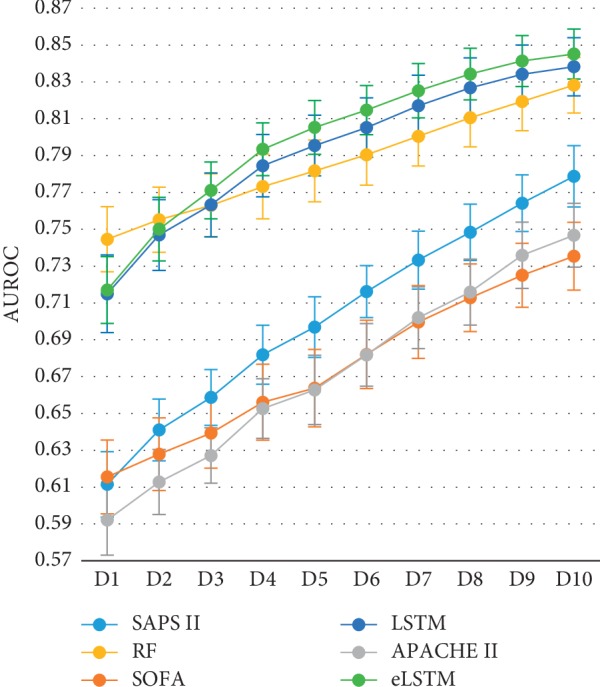
The AUROC values of all systems with data updating.

Figure 7 shows the time course of mortality prediction during one to ten days after the admission. It is seen that, with the available data updated daily, although the AUROC values of the various systems keep rising, through the whole procedure, the AUROC values of eLSTM, LSTM, and RF go higher than the three scoring systems. And from the third day, the eLSTM holds the highest value till the ending of the records. ANOVA followed by Dunnett test shows the AUROC value of the eLSTM model is significantly higher than that of LSTM and RF models (eLSTM vs. LSTM: *p*=0.011; eLSTM vs. RF: *p*=0.000). The charts also clearly reveal that while RF, LSTM, and the three scoring systems reach their highest performance on the last day, eLSTM achieves the corresponding levels at least 6 days earlier than the scoring systems and 2 and 1 days earlier than RF and LSTM, respectively. These facts demonstrate that eLSTM has stronger ability of dynamic prediction as well as early prognosis than the others.

**Figure 8:**
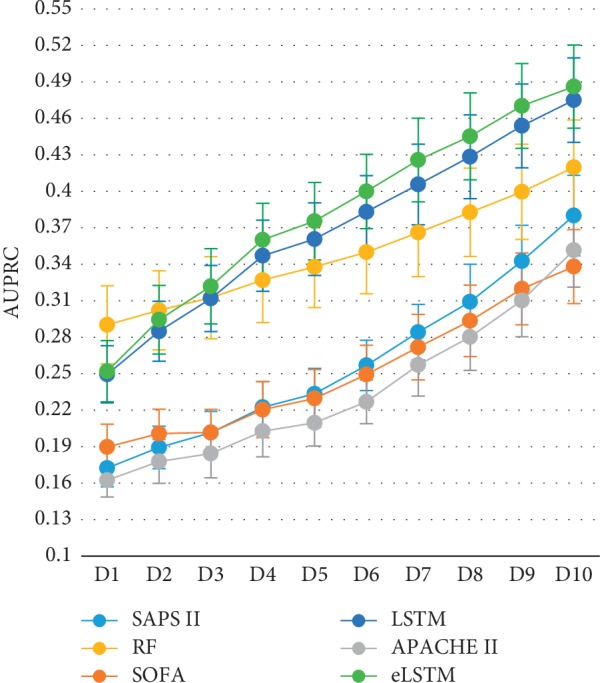
The AUPRC values of all systems with data updating.

Figure 8 shows that AUPRC has the similar trend with the data updating as AUROC. The eLSTM model harvests the largest AUPRC of 0.5 among all methods. ANOVA followed by Dunnett test exhibits that the AUROC value of eLSTM model is significantly higher than that of LSTM and RF (eLSTM vs. LSTM: *p*=0.043; eLSTM vs. RF: *p*=0.000).

### 3.3. Influence of the Number of LSTM Classifiers in eLSTM

**Figure 9:**
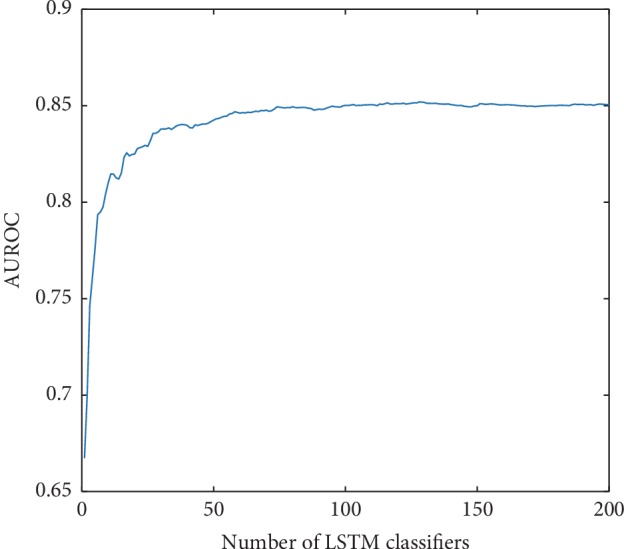
The AUROC values of eLSTM with the number of base LSTM classifiers increasing.

**Figure 10:**
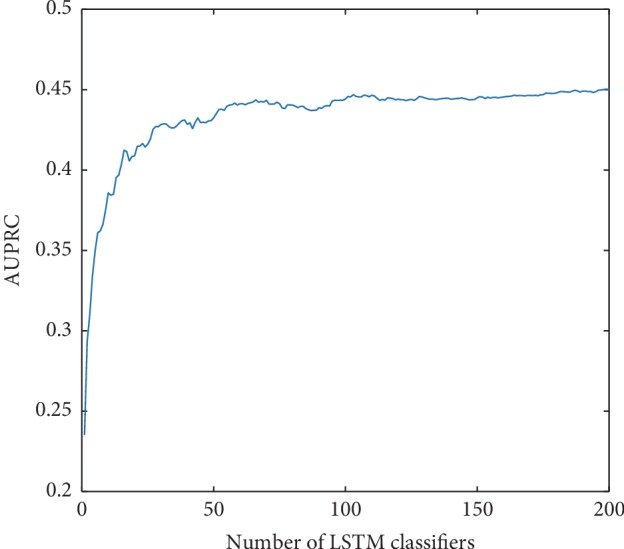
The AUPRC values of eLSTM with the number of base LSTM classifiers increasing.

The AUROC value of eLSTM goes up with the increase of the number of base LSTM classifiers (Figure 9). It has a steep ascent when less than 40 LSTM classifiers are integrated, then keeps a moderate rising, and finally stays at a plateau after 100 classifiers are involved. Similar situation is also observed in the AUPRC (Figure 10).

### 3.4. Influence of the Size of Variable Subset in eLSTM

**Figure 11:**
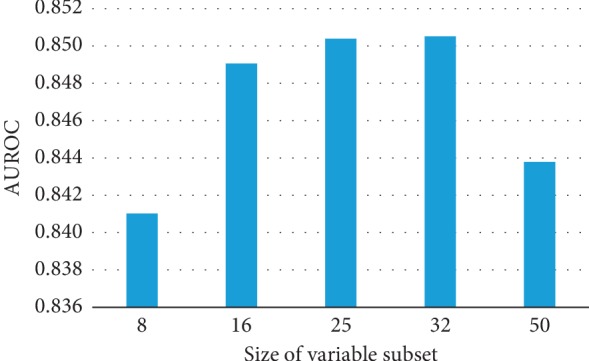
The AUROC values of eLSTM with multiple sizes of variable subset.

**Figure 12:**
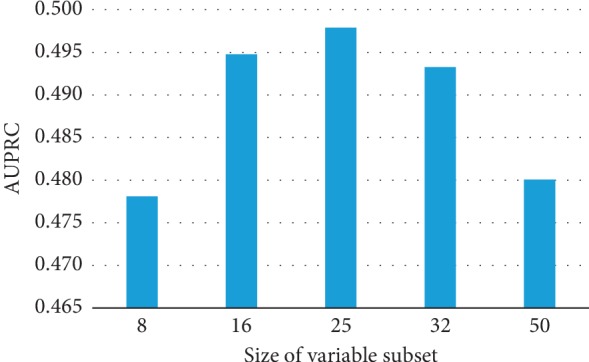
The AUPRC values of eLSTM with multiple sizes of variable subset.

ANOVA test indicates the size of variable subset in eLSTM models leads to significant difference in AUROC and as well as in AUPRC (AUROC: *F* = 45.932, *p*=0.000; AUPRC: *F* = 7.079, *p*=0.002). The AUROC values are similarly high for eLSTM with multiple sets of 16, 25, or 32 variables (Figure 11). And eLSTM achieves the largest AUPRC when the size of variable subset is 16, 25, or 32 (Figure 12). Pairwise comparison by Tukey test shows the AUROC and AUPRC values of eLSTM models trained by sets of 16, 25, and 32 variables are significantly higher than those of 8 and 50 variables (*p* < 0.05), while there are no significant differences among the models with sets of 16, 25, and 32 variables. In this work, the size of variable subset was set as the median value of 25, which is in agreement with the recommendation of literature [35].

## 4. Discussion

It is worth noticing that the algorithms of RF, LSTM, and eLSTM exhibit much better performance than the SAPS II, SOFA, and APACHE II scoring system (Table 2). It indicates that data-driven mathematical model may help improve the mortality prediction in ICU and further other clinical tasks. Different models serve different purposes and situations. The present work demonstrates that, in dynamic prediction, LSTM and eLSTM are superior to the RF algorithm. RF is commonly considered as an easy-to-use algorithm for decision making. However, it is not sensitive to time course, resulting in the weakness in exploiting temporal information in the series data. But in the LSTM block, the values in the previous time steps impose influence on the coming time steps; hence, the LSTM block is capable of capturing temporal trends of the data and suitable for time series modeling. Moreover, with the updating of the input data, the predicting ability of LSTM is continuously improved. In other words, LSTM has the advantage in dynamic predicting. The results demonstrate that generally, the eLSTM algorithm outperforms a single LSTM classifier. Also, it is seen in Figures 7 and 8 that the eLSTM model has much better achievement in early prediction than LSTM. It can be explained that instead of a single hypothesis space by one LSTM classifier, the eLSTM algorithm generates multiple base learners expanding the hypothesis space, which leads to a better approximation to the true hypothesis.

The proposed eLSTM algorithm successfully handles clinical time series data in ICU and provides a unified model for predicting the mortality of ICU patients. In ICU, patients are suffering from various diseases. Johnson et al. summarized the distribution of primary International Classification of Diseases (ICD) in the entire MIMIC-III database [44], as that the mostly common ones in ICU are infectious and parasitic diseases (ICD-9: 001–139), neoplasms of digestive organs, and intrathoracic organs, etc. (ICD-9: 140–239), endocrine, nutritional, metabolic, and immunity (ICD-9: 240–279), diseases of the circulatory system (ICD-9: 390–459), pulmonary diseases (ICD-9: 460–519), diseases of the digestive system (ICD-9: 520–579), diseases of the genitourinary system (ICD-9: 580–629), trauma (ICD-9: 800–959), and poisoning by drugs and biological substances (ICD-9: 960–979). Patients admitted to ICU are usually diagnosed with more than one kind of disease, i.e., syndrome. The physiological statuses of the patients are complex, and thus, it is difficult for a single learner to discover the patterns of the patients represented by recorded parameters. Thus, in previous relevant studies, the mathematical models in ICU were usually designed for single specific disease, such as heart failure or sepsis [49–53], and at present, it lacks universal quantitative mortality prediction approach covering all ICU patients. The diversity of the eLSTM is accomplished by employing bagging and RSM algorithm. In the construction of base learners, bootstrap sampling and RSM ensure the learners devoting to various patients and diseases. For model training, bootstrap sampling of ICU data produces divergent datasets of patients with different disease distributions. Meanwhile, RSM assembles different sets of physiological variables for representing patients' status. These procedures in training subsets broaden views at both instance and feature level of the ICU data and therefore yield dissimilar base LSTM classifiers. In this work, the setting of 25 variables in the model brings out the best performance (Figures 11 and 12). While too few variables would greatly decrease the base learner's classifying capacity, redundant variables would damage the learners' diversity. The result is consistent with the previous finding [35]. Moreover, as part of the bagging strategy at the output end of the model, individual base learners are integrated to make the ICU patients' general condition comprehensive and clear. Owing to individual learners' classifying capacity and the ensemble learning ability of the model, the proposed eLSTM algorithm is competent for capturing the complex relationship among the diseases and parameters in ICU data, thus enhancing the outcome prediction.

## 5. Conclusion

In this paper, we propose a new approach named eLSTM which can deal with the complex and heterogeneous ICU data for mortality prediction. The proposed eLSTM models obtain the prediction result by merging the results of multiple parallel LSTM classifiers. The base LSTM learners are trained on different subsets which are generated using bootstrapped samples and random feature subspace. Experimental results show that the proposed eLSTM algorithm effectively utilizes the ensemble framework in LSTM classifier and achieves excellent performance on the extracted MIMIC-III dataset. Also, it provides an early prognosis of ICU patients. The eLSTM model is promising to offer a universal quantitative tool for assessing risks of all patients in ICU and even for other complex clinical situations. In the future work, other approaches of aggregating component classifiers are worth investigating to optimize the structure as well as the algorithm.

## Figures and Tables

**Table 1:** Variables for mortality prediction.

Variable no.	Source table name	Variable name
1	LABEVENTS	BUN
2	LABEVENTS	WBC
3	LABEVENTS	HCO_3_^−^
4	LABEVENTS	Na^+^
5	LABEVENTS	K^+^
6	LABEVENTS	TBil
7	LABEVENTS	Plt
8	LABEVENTS	Cr
9	LABEVENTS	PH
10	LABEVENTS	HCT
11	LABEVENTS	Lactate
12	LABEVENTS	Hemoglobin
13	LABEVENTS	MCHC
14	LABEVENTS	MCH
15	LABEVENTS	MCV
16	LABEVENTS	Red Blood Cells
17	LABEVENTS	RDW
18	LABEVENTS	Chloride
19	LABEVENTS	Anion Gap
20	LABEVENTS	Glucose
21	LABEVENTS	Magnesium
22	LABEVENTS	Calcium, Total
23	LABEVENTS	Phosphate
24	LABEVENTS	INR
25	LABEVENTS	PT
26	LABEVENTS	PTT
27	LABEVENTS	Lymphocytes
28	LABEVENTS	Monocytes
29	LABEVENTS	Neutrophils
30	LABEVENTS	Basophils
31	LABEVENTS	Eosinophils
32	LABEVENTS	Base Excess
33	LABEVENTS	Calculated Total CO_2_
34	LABEVENTS	PCO_2_
35	LABEVENTS	Specific Gravity
36	LABEVENTS	ALT
37	LABEVENTS	AST
38	LABEVENTS	Alkaline Phosphatase
39	LABEVENTS	Albumin
40	LABEVENTS	PEEP
41	LABEVENTS	PaO_2_
42	CHARTEVENTS	GCS
43	CHARTEVENTS	SBP
44	CHARTEVENTS	HR
45	CHARTEVENTS	T
46	CHARTEVENTS	MAP
47	CHARTEVENTS	RR
48	CHARTEVENTS	A-aDO_2_
49	CHARTEVENTS	FiO_2_
50	LABEVENTS, CHARTEVENTS	PaO_2_/FiO_2_

**Table 2:** Evaluations of all mortality prediction systems (mean ± std).

	SAPS II	SOFA	APACHE II	RF	LSTM	eLSTM	ANOVA test
AUROC	0.7788 ± 0.0166^*∗*^	0.7354 ± 0.0184^*∗*^	0.7467 ± 0.0173^*∗*^	0.8282 ± 0.0151^*∗*^	0.8382 ± 0.0158^*∗*^	**0.8451** **±** **0.0136**	*F* = 926.328, *p*=0.000
AUPRC	0.3800 ± 0.0334^*∗*^	0.3381 ± 0.0307^*∗*^	0.3515 ± 0.0306^*∗*^	0.4197 ± 0.0393^*∗*^	0.4751 ± 0.0351^*∗*^	**0.4862** **±** **0.0345**	*F* = 426.683, *p*=0.000
Sensitivity/recall	0.6922 ± 0.0267^*∗*^	0.5418 ± 0.0394^*∗*^	0.6478 ± 0.0303^*∗*^	0.7197 ± 0.0395^*∗*^	0.7384 ± 0.0401^*∗*^	**0.7758** **±** **0.0321**	*F* = 438.869, *p*=0.000
Specificity	0.7404 ± 0.0102^*∗*^	**0.7958** **±** **0.0101**^*∗*^	0.7256 ± 0.0119^*∗*^	0.7807 ± 0.0218^*∗*^	0.7746 ± 0.0182^*∗*^	0.7503 ± 0.0136	*F* = 229.707, *p*=0.000
Accuracy	0.7347 ± 0.0096^*∗*^	0.7658 ± 0.0106^*∗*^	0.7164 ± 0.0113^*∗*^	**0.7734** **±** **0.0174**^*∗*^	0.7703 ± 0.0148^*∗*^	0.7533 ± 0.0112	*F* = 234.492, *p*=0.000
Precision	0.2633 ± 0.0145^*∗*^	0.2622 ± 0.0179^*∗*^	0.2404 ± 0.0149^*∗*^	**0.3063** **±** **0.0211**^*∗*^	0.3056 ± 0.0208^*∗*^	0.2941 ± 0.0158	*F* = 271.132, *p*=0.000
F1	0.3813 ± 0.0180^*∗*^	0.3532 ± 0.0227^*∗*^	0.3505 ± 0.0187^*∗*^	0.4290 ± 0.0216	**0.4317** **±** **0.0230**	0.4262 ± 0.0181	*F* = 363.817, *p*=0.000

^*∗*^The difference with the eLSTM model is significant at the 0.05 level. Bold indicates the highest mean value.

## Data Availability

The data used to support the findings of this study are available at MIMIC-III website (https://physionet.org/physiobank/database/mimic3cdb/).

## References

[1] Jin Y., Cai X. Y., Cai Y. C. (2012). To build a prognostic score model containing indispensible tumour markers for metastatic nasopharyngeal carcinoma in an epidemic area. *European Journal of Cancer*.

[2] Minne L., Abu-Hanna A., de Jonge E. (2009). Evaluation of SOFA-based models for predicting mortality in the ICU: a systematic review. *Critical Care*.

[3] Le Gall J.-R., Lemeshow S., Saulnier F. (1993). A new simplified acute physiology score (SAPS II) based on a European/North American multicenter study. *JAMA: The Journal of the American Medical Association*.

[4] Vincent J.-L., Moreno R., Takala J. (1996). The SOFA (Sepsis-related organ failure assessment) score to describe organ dysfunction/failure. *Intensive Care Medicine*.

[5] Knaus W. A., Draper E. A., Wagner D. P., Zimmerman J. E. (1985). APACHE II: a severity of disease classification system. *Critical Care Medicine*.

[6] Purushotham S., Meng C., Che Z., Liu Y. (2017). Benchmark of deep learning models on large healthcare MIMIC datasets. http://adsabs.harvard.edu/abs/2017arXiv171008531P.

[7] Johnson A. E., Pollard T. J., Mark R. G. Reproducibility in critical care: a mortality prediction case study.

[8] Lee J., Maslove D. M., Dubin J. A. (2015). Personalized mortality prediction driven by electronic medical data and a patient similarity metric. *PLoS One*.

[9] Luo Y., Xin Y., Joshi R., Celi L. A., Szolovits P. Predicting ICU mortality risk by grouping temporal trends from a multivariate panel of physiologic measurements.

[10] Kim S., Kim W., Park R. W. (2011). A comparison of intensive care unit mortality prediction models through the use of data mining techniques. *Healthcare Informatics Research*.

[11] Perotte A., Ranganath R., Hirsch J. S., Blei D., Elhadad N. (2015). Risk prediction for chronic kidney disease progression using heterogeneous electronic health record data and time series analysis. *Journal of the American Medical Informatics Association*.

[12] Breiman L. (2001). Random forests. *Machine Learning*.

[13] Caballero Barajas K. L., Akella R. Dynamically modeling patient’s health state from electronic medical records: a time series approach.

[14] Mikolov T., Karafiát M., Burget L., Černocký J., Khudanpur S. Recurrent neural network based language model.

[15] Du Y., Wang W., Wang L. Hierarchical recurrent neural network for skeleton based action recognition.

[16] Che Z., Purushotham S., Cho K., Sontag D., Liu Y. (2016). Recurrent neural networks for multivariate time series with missing values. http://adsabs.harvard.edu/abs/2016arXiv160601865C.

[17] Che Z., Purushotham S., Khemani R., Liu Y. Interpretable deep models for ICU outcome prediction.

[18] Choi E., Bahadori M. T., Schuetz A., Stewart W. F., Sun J. Doctor AI: predicting clinical events via recurrent neural networks.

[19] Sak H., Senior A., Rao K., Beaufays F. (2015). Fast and accurate recurrent neural network acoustic models for speech recognition. https://arxiv.org/abs/1507.06947.

[20] Hochreiter S., Schmidhuber J. (1997). Long short-term memory. *Neural Computation*.

[21] Tai K. S., Socher R., Manning C. D. (2015). Improved semantic representations from tree-structured long short-term memory networks. *Computer Science*.

[22] Faust O., Hagiwara Y., Hong T. J., Lih O. S., Acharya U. R. (2018). Deep learning for healthcare applications based on physiological signals: a review. *Computer Methods and Programs in Biomedicine*.

[23] Wang H., Yeung D. Y. (2016). Towards Bayesian deep learning: a survey. https://arxiv.org/abs/1604.01662.

[24] Sutskever I., Vinyals O., Le Q. V. Sequence to sequence learning with neural networks.

[25] Bahdanau D., Cho K., Bengio Y. (2014). Neural machine translation by jointly learning to align and translate. https://ui.adsabs.harvard.edu/abs/2014arXiv1409.0473B.

[26] Vinyals O., Toshev A., Bengio S., Erhan D. (2014). Show and tell: a neural image caption generator. http://adsabs.harvard.edu/abs/2014arXiv1411.4555V.

[27] Miotto R., Wang F., Wang S., Jiang X., Dudley J. T. (2017). Deep learning for healthcare: review, opportunities and challenges. *Briefings in Bioinformatics*.

[28] Lipton Z. C., Kale D. C., Elkan C., Wetzel R. (2015). Learning to diagnose with LSTM recurrent neural networks. http://adsabs.harvard.edu/abs/2015arXiv151103677L.

[29] Jo Y., Lee L., Palaskar S. (2017). Combining LSTM and latent topic modeling for mortality prediction. http://adsabs.harvard.edu/abs/2017arXiv170902842J.

[30] Harutyunyan H., Khachatrian H., Kale D. C., Galstyan A. (2017). Multitask learning and benchmarking with clinical time series data. http://adsabs.harvard.edu/abs/2017arXiv170307771H.

[31] Pham T., Tran T., Phung D., Venkatesh S. DeepCare: a deep dynamic memory model for predictive medicine.

[32] Awad A., Bader-El-Den M., McNicholas J., Briggs J. (2017). Early hospital mortality prediction of intensive care unit patients using an ensemble learning approach. *International Journal of Medical Informatics*.

[33] Ma T., Xiao C., Wang F. Health-ATM: a deep architecture for multifaceted patient health record representation and risk prediction.

[34] Dietterich T. G. Ensemble methods in machine learning.

[35] Ho T. K. (1998). The random subspace method for constructing decision forests. *IEEE Transactions on Pattern Analysis & Machine Intelligence*.

[36] Zhou Z.-H. (2012). *Ensemble Methods: Foundations and Algorithms*.

[37] Breiman L. (1996). Bagging predictors. *Machine Learning*.

[38] Nanni L., Lumini A., Brahnam S. (2012). A classifier ensemble approach for the missing feature problem. *Artificial Intelligence in Medicine*.

[39] Ozcift A., Gulten A. (2011). Classifier ensemble construction with rotation forest to improve medical diagnosis performance of machine learning algorithms. *Computer Methods and Programs in Biomedicine*.

[40] Özçift A. (2011). Random forests ensemble classifier trained with data resampling strategy to improve cardiac arrhythmia diagnosis. *Computers in Biology and Medicine*.

[41] Chen H., Yuan S., Jiang K. Wrapper approach for learning neural network ensemble by feature selection.

[42] Abreu P. H., Amaro H., Silva D. C. (2014). Overall survival prediction for women breast cancer using ensemble methods and incomplete clinical data. *XIII Mediterranean Conference on Medical and Biological Engineering and Computing 2013*.

[43] Kim H., Kim H., Moon H., Ahn H. (2011). A weight-adjusted voting algorithm for ensembles of classifiers. *Journal of the Korean Statistical Society*.

[44] Johnson A. E. W., Pollard T. J., Shen L. (2016). MIMIC-III, a freely accessible critical care database. *Scientific Data*.

[45] Bauer E., Kohavi R. (1999). An empirical comparison of voting classification algorithms: bagging, boosting, and variants. *Machine Learning*.

[46] Breiman L. (2001). Using iterated bagging to debias regressions. *Machine Learning*.

[47] Glorot X., Bengio Y. Understanding the difficulty of training deep feedforward neural networks.

[48] Twisk J., de Vente W. (2002). Attrition in longitudinal studies: how to deal with missing data. *Journal of Clinical Epidemiology*.

[49] Lagu T., Lindenauer P. K., Rothberg M. B. (2011). Development and validation of a model that uses enhanced administrative data to predict mortality in patients with sepsis. *Critical Care Medicine*.

[50] Vorwerk C., Loryman B., Coats T. J. (2009). Prediction of mortality in adult emergency department patients with sepsis. *Emergency Medicine Journal*.

[51] Steinhart B., Thorpe K. E., Bayoumi A. M., Moe G., Januzzi J. L., Mazer C. D. (2009). Improving the diagnosis of acute heart failure using a validated prediction model. *Journal of the American College of Cardiology*.

[52] Choi E., Schuetz A., Stewart W. F., Sun J. (2016). Using recurrent neural network models for early detection of heart failure onset. *Journal of the American Medical Informatics Association*.

[53] Fu X., Ren Y., Yang G. A computational model for heart failure stratification.

